# Kappa Free Light Chains in Cerebrospinal Fluid in Inflammatory and Non-Inflammatory Neurological Diseases

**DOI:** 10.3390/brainsci12040475

**Published:** 2022-04-03

**Authors:** Franz Felix Konen, Philipp Schwenkenbecher, Konstantin Fritz Jendretzky, Stefan Gingele, Torsten Witte, Kurt-Wolfram Sühs, Matthias Grothe, Malte Johannes Hannich, Marie Süße, Thomas Skripuletz

**Affiliations:** 1 Department of Neurology, Hannover Medical School, 30625 Hannover, Germany; konen.felix@mh-hannover.de (F.F.K.); schwenkenbecher.philipp@mh-hannover.de (P.S.); jendretzky.konstantin@mh-hannover.de (K.F.J.); gingele.stefan@mh-hannover.de (S.G.); suehs.kurt-wolfram@mh-hannover.de (K.-W.S.); 2 Department of Clinical Immunology & Rheumatology, Hannover Medical School, 30625 Hannover, Germany; witte.torsten@mh-hannover.de; 3 Department of Neurology, University Medicine Greifswald, 17475 Greifswald, Germany; matthias.grothe@uni-greifswald.de (M.G.); marie.suesse@uni-greifswald.de (M.S.); 4 Institute of Clinical Chemistry and Laboratory Medicine, University Medicine Greifswald, 17475 Greifswald, Germany; malte.hannich@med.uni-greifswald.de

**Keywords:** kappa-free light chains (KFLC), cerebrospinal fluid (CSF), Reiber’s diagram, neurological diseases, non-MS patients, biomarker

## Abstract

Background: Oligoclonal bands represent intrathecal immunoglobulin G (IgG) synthesis and play an important role in the diagnosis of multiple sclerosis (MS). Kappa free light chains (KFLC) are increasingly recognized as an additional biomarker for intrathecal Ig synthesis. However, there are limited data on KFLC in neurological diseases other than MS. Methods: This study, conducted at two centers, retrospectively enrolled 346 non-MS patients. A total of 182 patients were diagnosed with non-inflammatory and 84 with inflammatory neurological diseases other than MS. A further 80 patients were classified as symptomatic controls. Intrathecal KFLC production was determined using different approaches: KFLC index, Reiber’s diagram, Presslauer’s exponential curve, and Senel’s linear curve. Results: Matching results of oligoclonal bands and KFLC (Reiber’s diagram) were frequently observed (93%). The Reiber’s diagram for KFLC detected intrathecal KFLC synthesis in an additional 7% of the patient samples investigated (4% non-inflammatory; 3% inflammatory), which was not found by oligoclonal band detection. Conclusions: The determination of both biomarkers (KFLC and oligoclonal bands) is recommended for routine diagnosis and differentiation of non-inflammatory and inflammatory neurological diseases. Due to the high sensitivity and physiological considerations, the assessment of KFLC in the Reiber’s diagram should be preferred to other evaluation methods.

## 1. Introduction

In 2017, the McDonald criteria for multiple sclerosis (MS) were revised and a demonstration of the inflammation in cerebrospinal fluid (CSF) was included as a substitutional diagnostic criterion to proof dissemination in time [1]. In the past, the immunoglobulin G (IgG) index was considered as an important biomarker for the detection of intrathecal IgG synthesis [2]. The current gold standard for the detection of intrathecal IgG synthesis is the determination of oligoclonal bands [2,3]. In recent years, several other approaches to the automated biomarker determination have been investigated [4]. It was found that kappa free light chains (KFLC), which are a bystander product of Ig synthesis, have comparable diagnostic sensitivity to oligoclonal band analysis in MS patients [4,5,6,7,8]. Nephelometric, turbidimetric, or ELISA assays can be used for the automated determination of KFLC [4,9,10,11,12,13]. The most common approaches to determine KFLC in CSF are the calculation of the KFLC index (CSF/serum KFLC concentration / CSF/serum albumin concentration) and the use of CSF/serum albumin quotient (Qalb) dependent diagrams [4,5,7,8,14,15]. Although the KFLC allow quantitative interpretation compared with qualitative evaluation of oligoclonal bands, an excellent matching of results in MS patients has been reported [4]. Although intrathecal Ig synthesis has been reported in many neurological diseases, there are few studies to date that sufficiently describe the results of KFLC analysis in non-MS patients [4]. Two studies investigated KFLC in patients with neuroborreliosis and found moderate to high sensitivity of the KFLC interpretation method used [16,17]. Another study investigated KFLC concentrations in tick-borne encephalitis and found significant treatment effects [18]. Other studies aimed to investigate a KFLC-based threshold for discriminating between MS patients and patients with neuromyelitis optica spectrum disorders (NMOSD) [19,20,21]. In 2019, Reiber’s diagram for KFLC was published, which has since been shown to be the most sensitive and physiological approach for interpreting KFLC concentrations [4,5,6,15,21,22,23]. Nevertheless, studies of KFLC using the Reiber’s diagram in non-MS patients are rather rare [15,21,23]. 

In the present study, we therefore aimed to compare the diagnostic sensitivity and specificity of KFLC and oligoclonal band analysis in a large cohort of patients with different neurological diseases and symptomatic controls to establish the use of diagnostic biomarkers for intrathecal inflammation in different neurological diseases. The KFLC index of 5.9, Presslauer’s exponential curve, Senel’s linear function, and Reiber’s diagram for KFLC were used to interpret KFLC concentrations [7,8,14,15]. 

## 2. Materials and Methods

### 2.1. Patients

This retrospective, cross-sectional two-center study included a total of 346 patients who presented to the Department of Neurology at the Hannover Medical School (MHH) and the Department of Neurology at the University Medicine Greifswald (UMG) between 2008 and 2019. The CSF and serum pairs used were collected as part of routine diagnostics. Some of these patients had already been investigated previously with different foci [6,24,25]. Further patient information is depicted in Table 1. Patients were grouped by diagnosis, with 80 patients (23%) classified as symptomatic controls, as described by Teunissen et al., because these patients suffered from either unclassified headache, sensory disturbances, or dizziness [26]. However, no specific etiology that could have explained the patients’ symptoms was found [26]. A total of 182 (53%) patients suffered from non-inflammatory neurological diseases and 84 (24%) from inflammatory and infectious diseases of the central nervous system other than MS [26].

### 2.2. Analytical Procedures

All paired CSF and serum samples were analyzed in the Interdisciplinary CSF Laboratory of the UMG and the Neurochemistry Laboratory of the Department of Neurology of the MHH according to routine diagnostic procedures. The CSF cell count was performed manually using a Fuchs-Rosenthal counting chamber. The concentrations of albumin, IgG, IgM, and IgA in serum and CSF samples were determined by kinetic nephelometry (MHH: Beckman Coulter IMMAGE, Brea, CA, USA; UMG: ProSpec, Siemens Healthcare Diagnostics Products GmbH, Marburg, Germany). Reiber’s revised hyperbolic function was used to calculate the intrathecal synthesis of IgG, IgA, and IgM [27]. The determination of CSF-specific oligoclonal bands was performed by isoelectric focusing on polyacrylamide gels (EDC, Tübingen, Germany) followed by silver staining in the Neurochemistry Laboratory of the Department of Neurology of the MHH [28]. The oligoclonal bands were determined in the Interdisciplinary CSF Laboratory of the UMG by isoelectric focusing with a semiautomatic agarose electrophoresis system (Hydragel 9 CSF, Hydrasys 2Scan, Sebia GmbH, Fulda, Germany). Oligoclonal bands isolated in the CSF were classified as pathological and absent oligoclonal bands or oligoclonal bands in the CSF as well as in the serum were classified as normal or non-pathological.

For the determination of KFLC concentrations in CSF and serum samples by nephelometry, the N Latex FLC kappa Kit (Siemens Healthcare Diagnostics Products GmbH, Erlangen, Germany) was used on a BN Prospec analyzer (Siemens Healthcare Diagnostics Products GmbH, Erlangen, Germany) according to the manufacturer’s instruction. All sample pairs were stored at −80 °C after completion of the routine diagnostic work. KFLC were measured immediately after thawing. The pre-dilution of the CSF was set to 1:2; the pre-dilution of the serum was set to 1:100. The lower limit of quantification of the assay was 0.034 mg/L. According to the formulas described by Reiber et al., the hyperbolic reference range as well as the relative amount of intrathecally synthesized KFLC (KFLC IF) in relation to Qlim was calculated (discrimination line: Q_Kappa_(lim) = (3.27(Q_Alb_^2^ + 33)^0.5^ − 8.2) × 10^−3^; reference range: Q_Kappa_(mean) ± 3 CV (coefficient of variation) [15]. The exponential function of Presslauer et al. was defined by the subsequent formula: KFLC_IF_ = KFLC_Loc_/KFLC_CSF_ × 100 with KFLC_Loc_ = (KFLC_Ratio_ − KFCL_Lim_) × KFLC_Serum_, KFLC_Lim_ = 0.9358 × QAlb^0.6687^ and KFLC_Ratio_ = (KFLC CSF/KFLC Serum) [7]. For the linear function of Senel et al., the following formula was used: QKFLC = 14.85 + 2.41 × QAlb [8].

### 2.3. Statistical Analysis

GraphPad Prism (La Jolla, CA, USA; version 5.02) was used for the statistical analysis. The statistical significance level was set at 5%. The D’Agostino and Pearson omnibus normality test was used to test the normal distribution of the values. For intergroup comparison, the Mann–Whitney U-Test, the Kruskal–Wallis test, and the Friedman test with Dunn’s multiple comparison post hoc test were performed.

## 3. Results

Details of the clinical and CSF parameters of all included patients as well as the statistical significance of the differences between the groups can be found in Table 1. Overall, the interpretation of the oligoclonal bands and KFLC according to Reiber’s diagram revealed matching results in 93% (322) of the patient samples. A total of 11% (38) of the samples were oligoclonal band and KFLC positive, while 82% (284) were oligoclonal band and KFLC negative. However, in 24 (7%) of the patient samples, only the KFLC determination revealed pathological results suggestive of intrathecal Ig synthesis, while the oligoclonal band determination remained negative. These discordant findings were classified as “divergent” and are described below. 

### 3.1. Symptomatic Controls 

**Figure 1:**
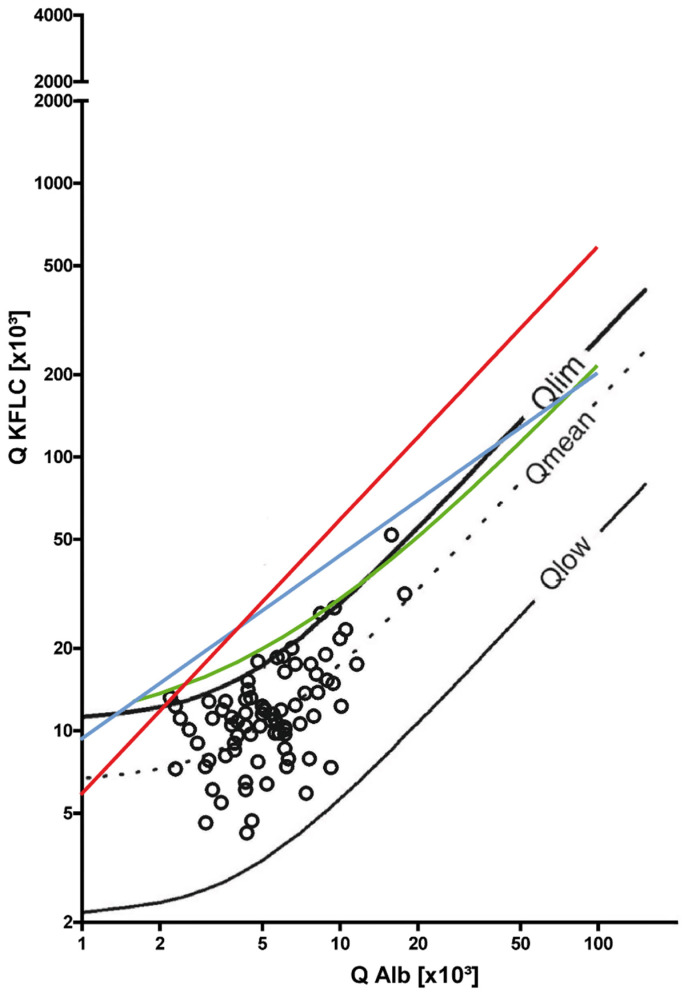
Oligoclonal bands and kappa-free light chain (KFLC) synthesis in the cohort of symptomatic controls. The black lines represent Reiber’s diagram for KFLC, the blue lines represent Presslauer’s KFLC exponential curve, the green lines represent Senel’s linear function, and the red lines represent the linear KFLC index of 5.9. All patients were oligoclonal band negative (black circles). Dots above the threshold lines represent positive patients for the method used. Depicted are patients suffering from unspecified headache, sensory disturbances, and dizziness, which were therefore considered symptomatic controls. QKFLC = KFLC quotient (KFLC in cerebrospinal fluid [mg/L]/KFLC in serum [mg/L]); Q Alb = albumin quotient (albumin in cerebrospinal fluid [mg/L]/albumin in serum [g/L]).

Details of the patients included in the symptomatic control cohort are shown in Figure 1 and Table 1. In the symptomatic controls cohort, no CSF-specific oligoclonal bands or an intrathecal IgG, IgA, and IgM synthesis, according to the Reiber’s diagrams, were observed in any patient sample. According to the Reiber’s diagram for KFLC, seven patient samples (9%) had a positive IF, indicating pathological results. 

Of these patients, sensory disturbances occurred in five patients and dizziness and unclassified headache in one patient each. In addition to a lack of intrathecal Ig synthesis, the CSF cell count as well as the age-adjusted CSF lactate concentration and QAlbumin were unremarkable in all patient samples, with the exception of one patient who had a mildly elevated QAlbumin. MRI was unremarkable in all patients and revealed only age-appropriate abnormalities. 

The interpretation of the KFLC concentrations according to the approaches of different authors revealed the following picture. According to Senel’s linear curve and the KFLC index > 5.9, one patient sample above the cut-off was identified in each case, which was also positive for KFLC according to Reiber’s diagram. In contrast, when applying Presslauer’s exponential function, no patient sample was classified as positive. 

### 3.2. Non-Inflammatory Neurological Diseases

**Figure 2:**
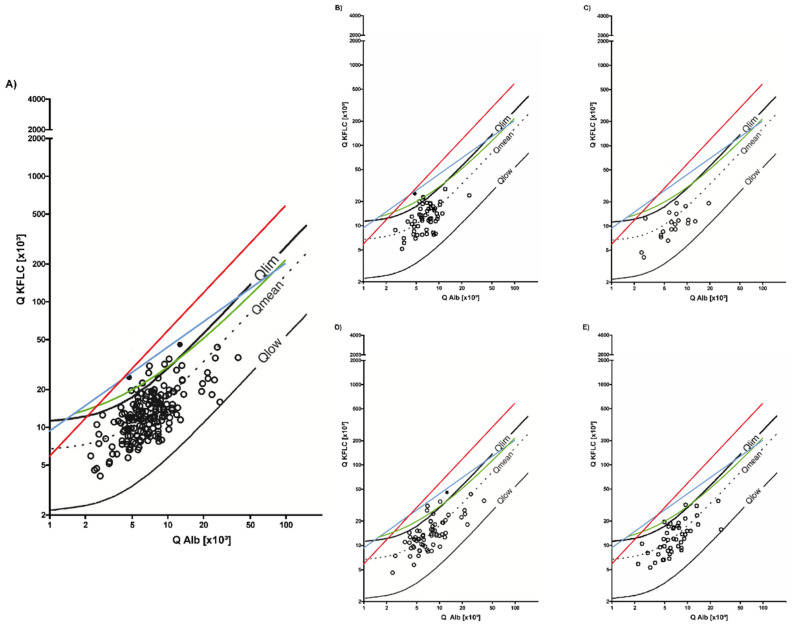
Oligoclonal bands and synthesis of kappa-free light chain (KFLC) in patients suffering from non-inflammatory neurological diseases. The black lines represent Reiber’s diagram for KFLC, the blue lines represent Presslauer’s KFLC exponential curve, the green lines represent Senel’s linear function, and the red lines represent the linear KFLC index of 5.9. The filled black dots represent patients with pathological oligoclonal band results (two patients), while the black circles represent oligoclonal band negative patients. Dots above the threshold lines represent positive patients for the method used. Depicted are all patients with non-inflammatory neurological diseases (**A**). Furthermore, oligoclonal band and KFLC results are shown for different disease entities (epilepsy (**B**), neurodegenerative diseases (Parkinson’s disease and dementia) (**C**), neuromuscular diseases (non-inflammatory peripheral nerve pathologies and amyotrophic lateral sclerosis (ALS)) (**D**), normal pressure hydrocephalus (NPH) or idiopathic intracranial hypertension (IIH) and non-inflammatory vascular diseases of the central nervous system (CNS) (**E**)). QKFLC = KFLC quotient (KFLC in cerebrospinal fluid [mg/L]/KFLC in serum [mg/L]), Q Alb = albumin quotient (albumin in cerebrospinal fluid [mg/L]/albumin in serum [g/L]).

Details of the patients with non-inflammatory neurological diseases are shown in Table 1 and Figure 2. Two patient samples (1%) showed isolated oligoclonal bands in the CSF. These patient samples also revealed positive results according to the Reiber’s diagram for KFLC. An intrathecal IgG, IgA, and IgM synthesis could not be detected in any sample using Reiber’s diagrams for Igs. A total of 10 patient samples (5%) showed intrathecal KFLC synthesis according to the Reiber’s diagram without intrathecal Ig synthesis. Deviating findings regarding the detection of intrathecal Ig synthesis according to oligoclonal bands and KFLC were made in a total of eight samples (4%). 

These patients suffered from ALS (*n* = 1), non-inflammatory polyneuropathy (*n* = 2), stroke (*n* = 2), and epilepsy (*n* = 3). The CSF cell count was below 4 cells/µL CSF in all but two samples (epilepsy and non-inflammatory polyneuropathy), while one patient sample revealed an elevated CSF lactate concentration (stroke). In five of these samples (ALS, *n* = 1; non-inflammatory polyneuropathy, *n* = 2, stroke, *n* = 1; epilepsy *n* = 1), an age-adjusted QAlbumin was elevated. MRI displayed age-appropriate abnormalities and characteristic pathology (stroke), but no inflammatory lesions were detected in any patient. 

Interpretation of KFLC concentrations according to the authors’ approaches showed that no patient sample was positive according to the Presslauer’s function or KFLC index of 5.9, while eight samples were positive according to Senel’s linear function. These eight patient samples were also positive according to Reiber’s diagram for KFLC.

### 3.3. Inflammatory Neurological Diseases

**Figure 3:**
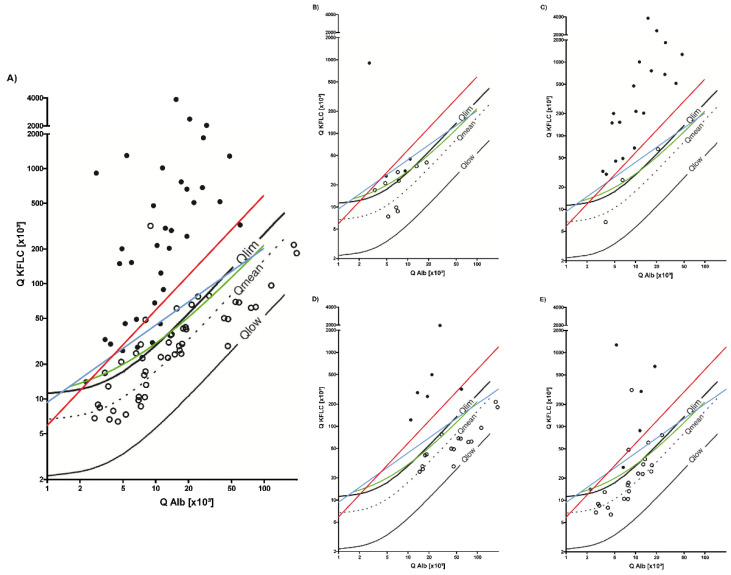
Oligoclonal bands and synthesis of kappa-free light chain (KFLC) in patients suffering from inflammatory neurological diseases. The black lines represent Reiber’s diagram for KFLC, the blue lines Presslauer’s KFLC exponential curve, the green lines Senel’s linear function, and the red lines the linear KFLC index of 5.9. The filled black dots represent patients with pathological oligoclonal band results, while black circles represent oligoclonal band negative patients. Dots above the threshold lines represent positive patients for the used method. Depicted are all patients suffering from inflammatory neurological diseases other than MS (**A**). Furthermore, oligoclonal band and KFLC results are shown for different disease entities (autoimmune-mediated encephalitis and vasculitis with affection of the central nervous system (CNS) (**B**), CNS infections with spirochetes (neuroborreliosis and neurosyphilis) (**C**), bacterial meningitis (**D**), and viral encephalitis (**E**)). QKFLC = KFLC quotient (KFLC in cerebrospinal fluid [mg/L]/KFLC in serum [mg/L]) and Q Alb = albumin quotient (albumin in cerebrospinal fluid [mg/L]/albumin in serum [g/L]).

The characteristics of the inflammatory neurological disease cohort are shown in Table 1, Table 2, and Figure 3. The prevalence of the oligoclonal bands was 42% (*n* = 35). Intrathecal IgG synthesis was found in 17 patient samples (20%), while 11 samples (13%) showed intrathecal IgA and 22 (26%) IgM synthesis. According to Reiber’s diagram for KFLC, 44 patients (52%) revealed pathological KFLC concentrations in the CSF, indicating intrathecal KFLC synthesis. All but two patient samples with intrathecal Ig synthesis according to the Reiber’s diagrams had pathological oligoclonal bands. One of these two patient samples had an isolated intrathecal IgM synthesis of 14% and positive KFLC results according to all interpretation approaches. The other patient had an isolated intrathecal IgA synthesis of 11% and a KFLC IF with close proximity to Q_Lim_. Both patients suffered from varizella zoster (VZV)-encephalitis. Thus, all samples but one with intrathecal Ig synthesis were positive for KFLC according to the Reiber’s diagrams. Regarding the detection of intrathecal Ig synthesis, 9 patient samples (11%) revealed divergent findings with non-pathological oligoclonal bands and increased KFLC IF. These patients were diagnosed with viral encephalitis (VZV, *n* = 3; Epstein-Barr-Virus (EBV), *n* = 1), neuroborreliosis (*n* = 1), neurosyphilis (*n* = 1), anti-NMDAR-encephalitis (N-methyl-D-aspartate-receptor, *n* = 2), and primary angiitis of the central nervous system (PACNS, *n* = 1). Age-adjusted QAlbumin was pathologically elevated in all samples, CSF cell count in all but two patient samples (anti-NMDAR-encephalitis), and CSF lactate concentration in only one sample (VZV-encephalitis). MRI was unremarkable with age-related structural changes in all patients except one who was diagnosed with PACNS and had typical MRI findings of cerebral artery irregularities, vessel wall enhancement, and white matter lesions. A precise estimation of the time span from symptom onset to sample collection could be retrospectively determined in four of these patients (9 days). The median time between the application of empirical anti-infective treatment and specimen collection was one day in all these patients with CNS infectious diseases. The anti-infective treatment consisted of ceftriaxone and acyclovir. In addition, corticosteroids were applied in one patient. In patients with inflammatory CNS diseases, rituximab (anti-NMDAR-encephalitis) and oral prednisolone (PACNS) were applied on the day of the sampling. In the treated patients with deviating results of oligoclonal band and KFLC determination, there were no significant differences in terms of KFLC concentration in the CSF compared to the other patients with the same disease entity.

KFLC determination revealed similar results when interpreted according to the KFLC index of 5.9 (*n* = 32/84, 38%) and Presslauer’s exponential function (*n* = 33/84, 39%). In contrast, more patient samples were KFLC-positive when using Senel’s linear function (*n* = 45/84, 54%). All patient samples that were positive according to Presslauer’s and Senel’s approaches and the KFLC index were also positive according to Reiber’s diagram for KFLC.

### 3.4. Sensitivity and Specificity of KFLC IF According to Reiber and Oligoclonal Band Determination to Distinguish Inflammatory and Non-inflammatory Diseases

For this analysis, the measurement results of symptomatic controls (*n* = 80) and samples with non-inflammatory neurological diseases (*n* = 182) were grouped to compare all patient samples with non-inflammatory neurological diseases (*n* = 262) and patient samples with inflammatory neurological diseases (*n* = 84). As shown in Table 3, determination of KFLC IF according to Reiber’s diagram has a higher sensitivity (52% versus 41% for oligoclonal band determination) but a lower specificity (94% versus 99%) for distinguishing between inflammatory and non-inflammatory neurological diseases. Both biomarkers have a very low negative predictive value (14% and 16%) while oligoclonal band determination has an excellent positive predictive value of 95% in comparison with the relatively low value for KFLC determination (72%).

## 4. Discussion

Since its publication in 2019, the Reiber’s diagram for KFLC has gained wide acceptance due to its consideration of the physiology of CSF flow and diffusion laws, leading several authors to recommend its use [4,5,6,15,21,22,23,24]. Our previous publications have again demonstrated the superior diagnostic sensitivity of the Reiber’s diagram in assessing intrathecal IgG synthesis in MS patients compared to alternative approaches (KFLC index, Senel’s linear function, Presslauer’s exponential function) [4,5,7,8,14,15]. 

In line with these results, the present study also demonstrated superior diagnostic sensitivity of the Reiber’s diagram for KFLC for detecting intrathecal Ig synthesis compared to alternative approaches in inflammatory neurological diseases other than MS. Across all disease entities and symptomatic controls, the KFLC index and Presslauer’s exponential function detected intrathecal Ig synthesis in 10% and Senel’s linear function in 16% of patient samples. Using the Reiber’s diagram for KFLC, Ig synthesis was detected in seven additional patient samples (18%). Recently, a linear cut-off value for the CSF KFLC concentration of 0.1 mg/dL was proposed as a substitute for the determination of oligoclonal bands in the diagnosis of MS [29]. Applying this cut-off, a low diagnostic sensitivity of about 79% was achieved for the diagnosis of MS, once again indicating the inferiority of linear cut-off values compared to Reiber’s diagram [29]. Furthermore, it should be considered that linear cut-off values do not take into account the physiology of CSF flow and the laws of diffusion. In addition, the interpretation of CSF KFLC concentrations should take into account the albumin quotient as an indicator of blood-CSF-barrier function. 

In general, excellent matching of oligoclonal band and KFLC results has been reported in MS patients, which was also observed in non-MS patients in the present study [4,5,6,15,21,22,23,24]. Nevertheless, aberrant results were found in a total of 7% of our patient samples. A total of 6% of symptomatic controls and non-inflammatory patient samples without oligoclonal bands revealed pathologic KFLC findings according to the Reiber’s diagram with close proximity to Q_Lim_ (median KFLC IF 46.7% in respect to QKFLC_mean_). Distinct renal function impairment or excessive serum KFLC concentrations as possible causes were not observed in these patients [25]. As a contribution of inflammatory mechanisms was not known in these patients, intrathecal KFLC synthesis is difficult to explain. It might be suggested that these KFLC values result from measurement inaccuracies, which is rather implausible as the measurement of KFLC is an automated procedure [6,15]. As shown before, the evaluation of KFLC in quotient diagrams results in a probability of 99.5% that a value above the upper reference limit is a consequence of an intrathecal KFLC synthesis [6]. On the other hand, the patient samples might have been wrongfully categorized, for example, as symptomatic control possibly leading to “false positive” results in these patients. However, in none of the included patients of the symptomatic controls, clinical, imaging- or CSF-based evidence for inflammatory processes could have been found. Since previous studies have reported a mean diagnostic specificity of 73% (53–100%) of the Reiber’s diagram for KFLC, it is rather implicated that there is a proportion of patient samples with KFLC synthesis of unknown cause in symptomatic controls and non-MS patients [4,5,6,15,21,22,23,24]. These patient samples may not be “false positive”, but were cases of inflammatory activity that could not have been detected by other biomarkers [15]. It has to be considered that oligoclonal bands as well as CSF KFLC are not disease specific but represent intrathecal Ig synthesis in general [30,31,32,33]. For example, a recent study demonstrated that pathological oligoclonal bands can be detected in up to 10% of patients with non-inflammatory neurological diseases, indicating intrathecal IgG synthesis [33]. In contrast to oligoclonal bands, intrathecal KFLC synthesis represents not only intrathecal IgG synthesis, but also synthesis of IgA and IgM [30]. It could be concluded that intrathecal IgA or IgM synthesis could be present in these patients, which was not detectable with Reiber’s diagrams for Igs. Due to the lack of CSF biomarker studies in the healthy population and the lack of association of intrathecal KFLC synthesis with direct B-cell activity in CSF, a definitive statement about the value of KFLC in CSF in non-inflammatory diseases is currently not possible. One possibility to further elucidate this issue might be to check the treatment response to immunomodulatory treatment in KFLC positive but oligoclonal band negative patients with neurological diseases in the future to assess for a diagnostic or predictive benefit of KFLC determination. A cross-sectional design as in the present study limits the capabilities of further investigating this issue, thus future studies should opt a prospective longitudinal study design with repeated lumbar punctures.

On the other hand, in 11% of the samples from inflammatory neurological diseases, intrathecal Ig synthesis was detected only by KFLC determination interpreted according to Reiber’s diagram, while no CSF specific oligoclonal bands were found. A total of 33% of patients with inflammatory neurological diseases with deviating findings (anti-NMDAR-encephalitis and PACNS) received immunomodulatory treatment (corticosteroids or rituximab) before the diagnostic procedure could be completed. In general, discrepancies between the findings of oligoclonal bands and KFLC concentrations are possible due to the use of corticosteroids and rituximab, so that divergent KFLC and oligoclonal band results can be suspected [30,34,35,36,37,38]. Transient non-pathological oligoclonal bands and unchanged pathological KFLC results according to Reiber’s diagram were reported in one patient using different immunomodulatory therapies [21]. Furthermore, the prevalence of pathological oligoclonal band results in patients with autoimmune-mediated encephalitis is dependent on other influencing factors. Suggested factors are the time of sampling during the course of the disease and the type of neuronal structure against which the antibodies are directed [39,40]. A total of 67% of the samples from inflammatory neurological diseases with intrathecal KFLC synthesis but non-pathological oligoclonal bands suffered from infectious diseases of the CNS (viral encephalitis, neuroborreliosis, and neurosyphilis). When infectious CNS disease is suspected, anti-infective treatment is often initiated before diagnostic lumbar puncture could be performed, as observed in our patients, which may suppress fulminant CNS infection [41,42]. It could be hypothesized that anti-infective treatment or sampling early in the course of the disease may have resulted in low inflammatory activity in the CNS in some of our patients, as evidenced by an increase in quantitatively detected inflammation parameters (KFLC), while qualitatively detected parameters (oligoclonal bands) remained normal. Furthermore, especially in samples from patients with infectious neurological diseases, it must again be taken into account that oligoclonal bands exclusively reflect intrathecal IgG synthesis [30]. In contrast, intrathecal KFLC synthesis also reflects intrathecal IgA and IgM synthesis [30]. As only intrathecal IgA or IgM synthesis may be present in various CNS infectious diseases, this is evidence that Ig synthesis in these patients might be missed with oligoclonal bands [43]. 

From a clinical point of view, both biomarkers are not suitable for the exclusion of inflammatory CNS diseases due to the very low negative predictive value. On the other hand, the non-inflammatory neurological disease cohort in the present study consisted mainly of patients with non-pathological oligoclonal bands, whereas the cohort of inflammatory diseases was characterized by many oligoclonal band positive patient samples, so that the high positive predictive value of oligoclonal band determination of 95% in the present study may be interpreted as a confounding factor. Nevertheless, in patients in whom intrathecal Ig synthesis is expected, KFLC could provide additional diagnostic sensitivity. Furthermore, in patients with uncertain CSF findings, repeated lumbar punctures could provide additional information and increase diagnostic accuracy. Tests for KFLC in CSF and serum should also be performed on pairs of samples taken in repeated lumbar punctures during follow-up. In general, it must be noted that a humoral immune response in the CSF analysis obtained by diagnostic lumbar puncture cannot always be expected in every inflammatory CNS disease. This means that the sensitivity of humoral biomarkers always depends on factors such as type of disease, time of lumbar puncture, and the treatment. This could be one reason for the comparatively low sensitivity of the investigated biomarkers in the inflammatory patient cohort. 

## 5. Conclusions

The determination of KFLC by using Reiber’s diagram and oligoclonal bands revealed predominantly matching results. The reasons for deviating results could be limited diagnostic specificity of both methods, the missing possibility of intrathecal IgA and IgM detection by oligoclonal band determination or immunosuppressive as well as anti-infective treatment. This needs to be further investigated. Due to its high sensitivity and physiological considerations, the evaluation of KFLC in the Reiber’s diagram offers advantages. Further multicenter studies should test the diagnostic performance of KFLC in different diseases and further investigate the different methods for determining a positive result.

## Figures and Tables

**Table 1:** Demographics and cerebrospinal fluid (CSF) parameters.

Characteristics	Symptomatic Controls, *n* = 80	Non-Inflammatory Neurological Diseases, *n =* 182	Inflammatory Neurological Diseases, *n* = 84	*p* Value
Females, *n* (%)	30/80 (38%)	95/182 (52%)	39/84 (46%)	<0.0001
Age [years], median (min–max)	40 (18–87)	61 (19–89)	48.5 (18–87)	0.0882
Oligoclonal bands, *n* (%)	0/80	2/182 (1%)	35/84 (42%)	<0.0001
Intrathecal IgG-synthesis, *n* (%)	0/80	0/182	17/84 (20%)	<0.0001
Intrathecal IgA-synthesis, *n* (%)	0/80	0/182	11/84 (13%)	<0.0001
Intrathecal IgM-synthesis, *n* (%)	0/80	0/182	22/84 (26%)	<0.0001
Reiber’s diagram for KFLC, *n* (%)	7/80 (9%)	10/182 (5%)	44/84 (52%)	<0.0001
KFLC index > 5.9, *n* (%)	1/80 (1%)	0/182	32/84 (38%)	<0.0001
KFLC index, mean (min–max)	2.3 (0.8–6)	2.2 (0.4–19.8)	21.5 (0.6–325.7)	<0.0001
KFLC CSF [mg/L], mean (min–max)	0.2 (0.04–0.7)	0.3 (0.03–2)	3.5 (0.03–35.2)	<0.0001
KFLC serum [mg/L], mean (min–max)	12.6 (5.6–53.2)	17.7 (4–95.2)	15.5 (3.7–73.8)	0.0767
Presslauer’s exponential curve, *n* (%)	0/80	0/182	33/84 (39%)	<0.0001
Senel’s linear curve, *n* (%)	1/80 (1%)	8/182 (4%)	45/84 (54%)	<0.0001
CSF cell count [cells/µL CSF], mean (min–max)	1 (0–4)	2 (0–15)	825 (0–14666)	<0.0001
CSF lactate concentration [mmol/L], mean (min–max)	1.7 (1.1–2.1)	2 (1.3–6.5)	3.5 (1.2–29.8)	< 0.0001
QAlbumin, mean (min–max)	5.8 (2.2–17.8)	7.6 (2.2–46.7)	21.9 (2.2–196.6)	< 0.0001
Diagnosis	Headache (unclassified); sensory disturbances; dizziness	Epilepsy (*n* = 54); IIH/NPH (*n* = 9); non-inflammatory peripheral nerve pathology (*n* = 52); non-inflammatory vascular diseases of the CNS (*n* = 35); ALS (*n* = 11); Parkinson’s disease (*n* = 9); dementia syndromes (*n* = 12)	Bacterial meningitis (*n* = 22); viral encephalitis (*n =* 27); neuroborreliosis (*n* = 17); neurosyphilis (*n* = 5); vasculitis with affection of the CNS (*n* = 5); autoimmune- mediated encephalitis (*n* = 8)	

IG = immunoglobulin, kappa-free light chains = KFLC, cerebrospinal fluid = CSF, CSF-serum albumin concentration quotient = QAlbumin, idiopathic intrathecal hypertension = IIH, normal pressure hydrocephalus = NPH, central nervous system = CNS, and amyotrophic lateral sclerosis = ALS. *p*-values below 0.05 are considered as statistically significant.

**Table 2:** Cerebrospinal fluid (CSF) parameters of inflammatory neurological disease patients.

Characteristics	Autoimmune-mediated Encephalitis, *n* = 8	Vasculitis with Affection of the CNS, *n* = 5	Viral Encephalitis, *n* = 27	Bacterial Meningitis, *n* = 22	Neuroborreliosis, *n* = 17	Neurosyphilis, *n =* 5
Oligoclonal bands, *n* (%)	1/8 (13%)	1/5 (20%)	6/27 (22%)	6/22 (27%)	15/17 (88%)	4/5 (80%)
Intrathecal IgG-synthesis, *n* (%)	1/8 (13%)	0/5	2/27 (7%)	4/22 (18%)	8/17 (47%)	2/5 (40%)
Intrathecal IgA-synthesis, *n* (%)	0/8	0/5	3/27 (11%)	2/22 (9%)	6/17 (35%)	0/5
Intrathecal IgM-synthesis, *n* (%)	2/8 (25%)	0/5	4/27 (15%)	2/22 (9%)	12/17 (71%)	2/5 (40%)
Reiber’s diagram for KFLC, *n* (%)	5/8 (63%)	2/5 (40%)	10/27 (36%)	6/22 (27%)	16/17 (94%)	5/5
KFLC index > 5.9, *n* (%)	1/8 (13%)	0/5	7/27 (26%)	5/22 (23%)	15/17 (88%)	4/5 (80%)
KFLC index, mean (min–max)	43.9 (1.4–325.7)	2.6 (1.2–4.3)	15 (1.3–242.3)	7.5 (0.3–69.2)	33.4 (1.8–116.1)	61.2 (3.8–254)
KFLC CSF [mg/L], mean (min–max)	1.3 (0.1–8.7)	0.6 (0.1–1.9)	2 (0.05–19.3)	3.3 (0.2–34.9)	6.7 (0.03–21.6)	7.9 (0.4–35.2)
KFLC serum [mg/L], mean (min–max)	11.4 (4.3–18.2)	22.3 (7.6–42.4)	18.2 (6.6–73.8)	13.6 (3.7–25)	13.5 (5.2–38.7)	15.8 (9.1–19.4)
Presslauer’s exponential curve, *n* (%)	1/8 (13%)	0/5	7/27 (26%)	6/22 (27%)	15/17 (88%)	4/5 (80%)
Senel’s linear curve, *n* (%)	5/8 (63%)	2/5 (40%)	10/27 (36%)	7/22 (32%)	16/17 (94%)	5/5
CSF cell count [cells/µL CSF], mean (min–max)	12 (1–36)	10 (1–40)	56 (7–304)	2951 (223–14666)	155 (0–1056)	13 (6–17)
CSF lactate concentration [mmol/L], mean (min–max)	1.6 (1.3–2.4)	1.8 (1.5–2.6)	2 (1.2–3.8)	8.2 (1.5–29.8)	2.2 (1.3–3.9)	1.7 (1.5–2)
QAlbumin, mean (min–max)	6.6 (2.8–13.5)	9.8 (5.2–18.8)	9.5 (2.2–24.2)	53 (11–196.6)	16.1 (3.4–47.3)	7.3 (3.8–15.2)

Central nervous system = CNS, IG = immunoglobulin, kappa-free light chains = KFLC, cerebrospinal fluid = CSF, and CSFserum albumin concentration quotient = QAlbumin.

**Table 3:** Sensitivity and specificity of the kappa-free light chains (KFLC) determined with the formulae described by Reiber et al. and determination of the oligoclonal bands.

	Inflammatory Diseases *n* = 84	Non-Inflammatory Diseases *n* = 262	*n* = 346
QKFLC > Qlim (KFLC)	*n* = 44	*n* = 17	PPV 72%
Oligoclonal bands positive	*n* = 35	*n* = 2	PPV 95%
QKFLC < Qlim (KFLC)	*n* = 40	*n* = 245	NPV 14%
Oligoclonal bands negative	*n* = 49	*n* = 260	NPV 16%
	Sens KFLC 52% Sens OCB 41%	Spec KFLC 94% Spec OCB 99%	

Qlim = curve for the upper limit of the QKFLC reference range, QKFLC = CSF-serum concentration quotient of kappa-free light chains, positive predictive value = PPV, negative predictive value = NPV, sensitivity = sens, specificity = spec, and oligoclonal bands = OCB.

## Data Availability

The datasets used and/or analyzed during the current study are available from the corresponding author on reasonable request.
